# Wasserstein distributional distance and linear programming convergence planning as monitoring network benchmarking tools: cross-continental surface water quality assessment across Brazilian and European river basins

**DOI:** 10.1007/s10661-026-15914-w

**Published:** 2026-09-16

**Authors:** Hugo Pimentel Tavares, Nilo Antônio de Souza Sampaio

**Affiliations:** Faculdade de Tecnologia (FAT), Departamento de Engenharia Ambiental (DEAMB), Universidade do Estado do Rio de Janeiro (UERJ), Resende, 27537-000 RJ Brazil

**Keywords:** Monitoring network benchmarking, Optimal transport distance, Wasserstein distance, TOPSIS, Linear programming, Water quality assessment, Pollution monitoring, Cross-continental comparison, Convergence planning

## Abstract

Evaluating the performance of long-term water quality monitoring networks against international reference systems requires analytical tools capable of comparing entire empirical distributions — not merely summary statistics — yet distributional benchmarking methods have rarely been applied at the cross-continental scale. This study develops and applies a four-method analytical framework — combining Wasserstein-1 distributional distance (earth mover’s distance), Shannon-entropy-weighted TOPSIS, linear programming (LP) convergence planning, and principal component analysis with hierarchical cluster analysis (PCA/HCA) — to compare ten São Paulo river basins (Brazil) against 23 European countries monitored under the Water Framework Directive (WFD), using a harmonised dataset of 646,356 CETESB records and 998,410 GRQA v1.4 records covering seven common physicochemical parameters over a shared window of 2016–2022. The Wasserstein-1 analysis reveals that the distributional gap is non-uniform: Afluentes do Paraíba do Sul (composite $W_1 = 0.245$) and the Cantareira system ($W_1 = 0.333$) are already within the distributional range of lower-ranking European countries, while the Alto Tietê ($W_1 = 2.060$) and Médio Tietê ($W_1 = 1.392$) are distributional outliers. Shannon-entropy-weighted TOPSIS ranking of the 20 entities with complete parameter coverage (all ten Brazilian basins and the ten European countries with complete five-parameter medians) places Afluentes-PSB seventh overall (score = 0.901), above Portugal, Poland, Germany, and Belgium, while the Alto Tietê (0.246) and Médio Tietê (0.194) rank last. Mann–Whitney U tests confirm statistically significant distributional differences for six of seven parameters, with large Cliff’s δ for temperature (δ=+0.858), dissolved oxygen (δ=-0.768), and biochemical oxygen demand (δ=+0.571); nitrate is the sole parameter with no significant difference (p=0.354; δ=-0.010, negligible). The LP convergence model, constrained by historically observed maximum improvement rates in the CETESB dataset, identifies dissolved oxygen as the binding convergence constraint for seven of ten basins, with critical-path convergence times ranging from 4.4 years (Afluentes-PSB) to 14.8 years (Alto Tietê) under the 90th-percentile feasibility assumption, lengthening to 5.5 and 28.7 years respectively under a more conservative 75th-percentile assumption; total phosphorus carries the highest LP cost weight (w=0.669) but is the rate-limiting parameter for only one basin (Tietê-Médio). Rankings are robust to the weighting scheme (Kendall’s $\tau \ge 0.86$ against equal-weight and CRITIC alternatives). Applied externally to 33 Polish and 26 Italian sub-basins under a leave-one-country-out design, basin scores correlate negatively with an independent measure of anthropogenic pressure in both countries (ρ=-0.44 and -0.42), indicating that the benchmarking and ranking components transfer to monitoring systems on which the framework was not developed. PCA/HCA distinguishes four typological clusters: the Tietê metropolitan arc (Cluster 2), a predominantly European mainstream (Cluster 1), a clean-reference group (Cluster 3) comprising four Brazilian basins together with Portugal, and an intermediate-impaired group (Cluster 4). These findings demonstrate that São Paulo’s monitoring network is not uniformly distant from the European reference; the proposed framework provides a replicable, distribution-sensitive KPI for monitoring network performance assessment applicable to any national network linked to an international reference archive.

**Supplementary Information:**

The online version contains supplementary material available at https://doi.org/10.1007/s10661-026-15914-w.

## Introduction

Long-term water quality monitoring networks generate vast empirical records whose full informational content is rarely exploited in comparative assessments. Standard analytical practice reduces multi-year, multi-station distributions to point summaries — means, medians, compliance rates — discarding the distributional shape, inter-quantile structure, and tail behaviour that are most relevant for pollution risk assessment and monitoring network evaluation (Jung et al., 2020; Rozemeijer et al., 2025). This information loss is particularly consequential when the objective is to benchmark a national monitoring network against an international reference: two networks with identical medians may differ profoundly in their distributional architecture, and only distributional metrics can reveal this divergence.

This study addresses that gap by applying Wasserstein-1 distance — the earth mover’s distance from optimal transport theory (Villani, 2009) — as a distributional benchmarking metric for surface water quality monitoring networks. The Wasserstein distance quantifies the minimum cost of transforming one empirical distribution into another, capturing differences in location, spread, skewness, and tail behaviour simultaneously. Complementing this distributional analysis, a linear programming (LP) convergence model translates Wasserstein gaps into actionable, parameter-specific improvement targets constrained by historically observed remediation rates — providing monitoring agencies with a prioritisation framework that is grounded in the monitoring record itself rather than engineering hypotheticals (Ying et al., 2025).

The framework is demonstrated on two monitoring networks of exceptional density and longevity. The Brazilian network, operated by CETESB — the São Paulo State Environmental Company — has recorded 646,356 physicochemical measurements across ten river systems since 1978, constituting one of the largest long-term surface water quality archives in the Global South. The European reference network is provided by the Global River Water Quality Archive (GRQA v1.4) (Virro et al., 2025), which aggregates 998,410 harmonised records from 23 countries monitored under the Water Framework Directive (WFD; Directive 2000/60/EC) (European Parliament and Council, 2000). The WFD network constitutes the world’s largest standardised transnational monitoring system and provides the most appropriate international benchmark against which a Southern Hemisphere long-term network can be evaluated. The São Paulo network is particularly suitable as a demonstration case because it encompasses contrasting basin typologies — from severely impaired metropolitan systems to near-pristine headwaters — that stress-test the full range of the benchmarking methodology (Bandeira de Almeida et al., 2023). Prior analyses of this network have documented severe chronic impairment in the metropolitan Tietê corridor and quantified detection latency under multivariate statistical process control when benchmarked against eight European countries (Pimentel Tavares & de Souza Sampaio, 2026c), motivating the present distributional approach as a complement to monitoring-efficiency studies.

The analytical gap that motivates this study is methodological. Existing cross-continental comparisons typically compare mean or median values — a summary statistic that discards distributional shape, ignores within-basin variability, and cannot distinguish whether two populations with the same median have structurally similar or dissimilar distributions. Wasserstein distance (the earth mover’s distance from optimal transport theory) (Villani, 2009) resolves this limitation by measuring the minimum cost of transforming one empirical distribution into another, thereby capturing differences in location, spread, skewness, and tail behaviour simultaneously. To our knowledge, this is the first application of Wasserstein distance to cross-continental river water quality benchmarking.

The complementary LP convergence model addresses a distinct but related gap. Knowing the current distributional distance is informative; knowing the minimum intervention required to close it — and which parameter constitutes the binding constraint — is actionable. By anchoring feasibility constraints to historically observed improvement rates in the CETESB dataset, the LP model provides basin-specific convergence timelines that are grounded in empirical evidence rather than normative assumptions.

This study pursues five objectives: (i) to quantify the Wasserstein-1 distributional distance between each São Paulo monitoring basin and the European reference distribution for seven physicochemical parameters over the shared window 2016–2022; (ii) to rank the monitored entities with complete parameter coverage (10 Brazilian basins and the 10 European countries with complete five-parameter medians) using Shannon-entropy-weighted TOPSIS on a common parameter set; (iii) to test and quantify distributional differences using Mann–Whitney U statistics and Cliff’s δ effect size; (iv) to formulate and solve a linear programme identifying minimum-cost convergence targets and critical-path timelines per monitoring basin; and (v) to characterise the typological structure of both monitoring networks via PCA and Ward hierarchical cluster analysis, positioning Brazilian basins within the European quality landscape.

## Literature review

### Long-term water quality dynamics in Brazilian river basins

The challenge of translating regulatory frameworks into measurable water quality improvement is not unique to Brazil: evidence from China’s seven major river basins shows significant long-term improvement alongside persistent spatial heterogeneity between southern and northern rivers despite centralised quality targets (Fu et al., 2023); Mediterranean catchments under the WFD face similar compliance gaps (European Parliament and Council, 2000); and India’s Ganga basin governance reforms have produced mixed results across monitoring networks. The São Paulo case is analytically tractable precisely because of the exceptional density and continuity of the CETESB monitoring record — making it a benchmark for evaluating governance performance in long-term monitored systems worldwide.

The literature on Brazilian river water quality reveals a persistent structural tension between regulatory ambition and monitored outcomes. The National Water Resources Policy (Lei 9.433/1997) and CONAMA Resolution 357/2005 (CONAMA, 2005) established a sophisticated governance architecture that has been extensively studied but has produced spatially heterogeneous outcomes. Bandeira de Almeida et al. (Bandeira de Almeida et al., 2023) analysed 56 São Paulo monitoring sites across four watersheds (2004–2018) and identified that the parameter set required for regulatory compliance monitoring differs substantially from that required for trend detection, highlighting a systematic mismatch between monitoring design and management objectives.

In the Paraíba do Sul basin — shared among Rio de Janeiro, São Paulo, and Minas Gerais states — multi-agency integration across 345,942 records (1977–2025) has revealed that microbiological contamination from untreated domestic sewage remains the dominant constraint on regulatory compliance (overall conformity = 23.3% for thermotolerant coliforms under CONAMA Class 2), while nitrate has exhibited the strongest long-term upward trend ($R^2 = 0.594$; β=+0.027 mg L$^{-1}$ yr$^{-1}$), indicating an emerging agricultural loading signal that overlaps with the Cantareira and Piracicaba catchments of the present study (Pimentel Tavares et al., 2026).

The Alto Tietê sub-basin, encompassing the São Paulo Metropolitan Region, has been documented as among the most severely impaired river systems in the Global South, with dissolved oxygen approaching zero at multiple monitoring points in the urban core (Bandeira de Almeida et al., 2023; Cunha et al., 2013). The downstream Médio and Baixo reaches partially recover through dilution and photosynthetic reoxygenation, but carry elevated nutrient loads that drive downstream eutrophication pressure in the Paraná River system.

### Water quality monitoring network benchmarking and the water framework directive

The WFD (European Parliament and Council, 2000) introduced ecological status classification for European surface water bodies, requiring member states to achieve at least “good ecological status” by 2027. Despite 25 years of implementation, only about 37% of European surface water bodies had reached good or high ecological status over the 2015–2021 reporting cycle (European Environment Agency, 2024), illustrating that even the world’s most resourced governance architecture faces structural implementation gaps. This context is important: benchmarking against Europe is not benchmarking against perfection, but against a heterogeneous system with its own gradient of quality and governance capacity.

Cross-continental water quality comparison has traditionally relied on index-based summaries. Tsakiris et al. (2015) developed a multi-criteria framework for evaluating water scarcity measures across Mediterranean and non-Mediterranean contexts, demonstrating that ranking of management options is highly sensitive to the choice of criteria weights. Simian et al. (2025) compared 22 water quality indices across multiple datasets in a *Water Resources Management* study, showing that index selection introduces systematic bias in entity ranking and advocating for ensemble or multi-criteria approaches. Recent advances in water quality index frameworks have further highlighted the sensitivity of comparative assessments to parameter selection and the need for uncertainty-aware approaches (Poursaeid, 2025; Singh et al., 2026). The present study contributes to this agenda by replacing index-based comparison with distributional distance analysis, removing the aggregation step that discards information about distributional shape.

A parallel and rapidly growing body of work approaches water quality uncertainty from the direction of predictive modelling, and it is worth setting out precisely how the present framework differs from it, since the two families are complementary rather than competing. Liang et al. (2025) combine multi-source remote sensing and in-situ data with machine learning to estimate water quality parameters in urban rivers; Wan et al. (2022) couple deep learning with feature extraction to predict non-point-source pollution; Alizamir et al. (2025) develop an LSTM–CNN architecture for fluorescent dissolved organic matter and use SHAP values to make the resulting predictions interpretable; and Poursaeid (2025) pair artificial-intelligence simulators with metaheuristic optimisers to propagate uncertainty through water quality instability scenarios. These studies share three features: the object of inference is a *future or unobserved value*; uncertainty is a property of the fitted model, quantified through ensembles, residual distributions, or attribution measures; and feature engineering and optimiser selection are central design choices because they govern predictive skill. The framework developed here inverts each of these. The object of inference is the *observed empirical distribution itself*, compared against a reference distribution without any fitted model interposed; there is consequently no model uncertainty to propagate, and no optimiser in the learning sense — the only optimisation is the linear programme, which allocates improvement effort rather than fitting parameters. Uncertainty enters instead through the analytical assumptions, and is addressed by systematically varying them: the weighting scheme, the benchmark stringency, and the feasibility percentile are each perturbed and the conclusions re-examined (“Discussion” section). The two approaches thus answer different questions — what a monitoring network *will* record next, versus how far what it *has* recorded stands from an international reference — and a natural direction for future work is to use distributional distance as the target variable for the predictive architectures cited above. We note that the analysis is scoped to seven physicochemical and general pollution indicators shared by both monitoring networks; biological quality elements and emerging contaminants, which are important for comprehensive ecological assessment, are not included and represent a known limitation discussed further in the “Discussion” section.

### Optimal transport distance in water resources analysis

Optimal transport (OT) theory, originally formalised by Monge (1781) and placed on rigorous mathematical foundations by Kantorovich (1942), has seen rapid adoption in environmental data science since Villani’s definitive treatment (Villani, 2009). The Wasserstein-*p* distance between probability measures μ and ν is defined as:

1 $$\begin{aligned} W_p(\mu ,\nu ) = \left( \inf _{\gamma \in \Gamma (\mu ,\nu )} \int _{\mathcal {X}\times \mathcal {X}} d(x,y)^p \,\textrm{d}\gamma (x,y)\right) ^{1/p} \end{aligned}$$

where $\Gamma (\mu ,\nu )$ is the set of all couplings of μ and ν, and $d(\cdot ,\cdot )$ is a ground metric on the sample space $\mathcal {X}$. For univariate distributions and p=1 (the earth mover’s distance), Eq. (1) simplifies to the analytically tractable form:

2 $$\begin{aligned} W_1(\mu ,\nu ) = \int _0^1 \bigl |F_\mu ^{-1}(t) - F_\nu ^{-1}(t)\bigr | \,\textrm{d}t \end{aligned}$$

where $F^{-1}$ denotes the quantile function of the respective distribution. This formulation is computationally advantageous for empirical distributions: $W_1$ reduces to the $L^1$ norm of the difference between empirical quantile functions, which is computable in O(nlogn) time via sorted-array operations.

Applications of optimal-transport distance to environmental and signal-processing problems have grown since Kolouri et al. (Kolouri et al., 2017) reviewed optimal mass transport for signal processing and machine learning, including the sliced Wasserstein distance for high-dimensional comparison. In the water-quality domain, however, distributional distances remain far less common than moment- or index-based comparisons. To our knowledge, the present study is the first to apply Wasserstein distance as a cross-continental ben-chmarking metric for river water quality at basin scale.

### Multi-criteria decision making methods in water quality management

Multi-criteria decision making (MCDM) has become a standard analytical tool in water resources management (Simian et al., 2025; Tsakiris et al., 2015). The Technique for Order of Preference by Similarity to Ideal Solution (TOPSIS), introduced by Hwang and Yoon (1981), ranks alternatives by their relative proximity to a positive ideal solution and distance from a negative ideal, providing a transparent and computationally tractable ranking that has been widely validated in comparative resource management contexts. A critical methodological issue is the assignment of criterion weights: subjective weighting introduces analyst bias that can substantially alter rankings (Kolios et al., 2016).

Shannon entropy weighting (Shannon, 1948) resolves this by deriving weights objectively from the informational content (spread) of each criterion column in the decision matrix. Criteria exhibiting higher variability across alternatives receive higher weights, under the premise that more discriminating criteria deserve greater influence. This approach has been applied to water quality ranking in several recent studies (Feng et al., 2019) but has not previously been combined with Wasserstein-distance analysis for cross-continental comparison. Tavares and de Souza Tavares and de Souza Sampaio (2026) demonstrated the methodological compatibility of LP and entropy-weighted TOPSIS in a multi-country energy systems comparison, providing the methodological precedent for the adaptation applied here to river water quality. Recent applications of TOPSIS in cooperative water allocation contexts (Janjua et al., 2025) further confirm the method’s tractability in complex water resources planning.

### Linear programming for environmental convergence planning

LP has a long history in water resources management for allocation and planning problems (Loucks & van Beek, 2017). Its application to *convergence planning* — determining the minimum intervention vector required to achieve a specified target state within feasibility constraints — has been used in atmospheric pollution reduction planning (Nagurney & Toyasaki, 2003) and fisheries management (Grafton et al., 2000), but has rarely been formalised for river water quality. Pimentel Tavares and de Souza Sampaio (2026b) applied an integrated MCDA–LP framework to prioritise 347 EPA monitoring stations in California, demonstrating a 36.9% improvement over uniform allocation and establishing the methodological basis for LP-driven network optimisation in water quality assessment; the present study extends this approach to cross-continental distributional convergence planning. Similarly, Pimentel Tavares and de Souza Sampaio (2026a) applied a mixed-integer LP framework to rationalise the Paraíba do Sul monitoring network, achieving a 65.6% station reduction while maintaining full river coverage — a complementary network design application on the same CETESB dataset used here. Ward (2025) identifies optimal allocation of remediation investments across basins as a central unresolved challenge in global water quality management, suggesting that efficiency-frontier analysis tools are needed. The LP formulation developed here directly addresses this need by combining observed distributional gaps (from the Wasserstein analysis) with empirically estimated feasibility constraints (from the CETESB historical record) to generate minimum-effort convergence plans for each basin.

### PCA and cluster analysis for typological classification

Principal component analysis and hierarchical cluster analysis are standard tools for characterising multivariate structure in water quality datasets (Helsel et al., 2020). Their application here extends beyond a single monitoring network to a cross-continental entity set, allowing Brazilian basins to be positioned within the natural typological structure of the 20-entity distribution. The identification of which cluster archetype each Brazilian basin belongs to has direct managerial implications: a basin co-clustering with clean Northern European systems requires a fundamentally different governance response than one clustering with heavily impaired Central European rivers.

## Materials and methods

### Monitoring network data sources and harmonisation

Two primary datasets were used. The Brazilian dataset comprises 646,356 physicochemical records from 141 CETESB monitoring stations distributed across ten river systems in São Paulo State and the São Paulo–Minas Gerais border (January 1978 to April 2026). The ten river systems are: (1) Rio Paraíba do Sul (main channel), (2) Afluentes do Paraíba do Sul — UGRHI 2, (3) Rio Piracicaba, (4) Rio Capivari, (5) Rio Jundiaí, (6) Sistema Cantareira — Afluentes UGRHI 5, (7) Rio Tietê — Alto Tietê (UGRHI 6), (8) Rio Tietê — Médio Tietê (UGRHI 10), (9) Rio Tietê — Baixo Tietê (UGRHI 13), and (10) Rio Mogi-Guaçu.

São Paulo State covers approximately 248,000 km$^2$ in southeastern Brazil and spans a marked physiographic gradient. The eastern margin is dominated by the Serra do Mar and Serra da Mantiqueira escarpments, where elevations exceed 2000 m and remnants of Atlantic Forest persist; the interior consists of the gently inclined Paraná sedimentary basin, falling to below 400 m in the west. The climate is humid subtropical to tropical (Köppen Cfa and Cwa), with a wet summer from October to March delivering the majority of the 1200–1800 mm annual precipitation and a dry winter that concentrates pollutant loads under reduced dilution. Two contrasting drainage systems are represented. The Tietê rises in the coastal range and flows inland across the state to the Paraná River, passing through the São Paulo Metropolitan Region (∼22 million inhabitants) in its upper reach; the Paraíba do Sul and its UGRHI 2 tributaries drain the Mantiqueira–Serra do Mar corridor toward Rio de Janeiro State. Land use ranges from dense urban and industrial occupation in the Alto Tietê and the Piracicaba–Capivari–Jundiaí corridor, through extensive sugarcane, citrus, and pasture in the Mogi-Guaçu and Médio Tietê catchments, to predominantly forested headwaters in the Cantareira system and the Afluentes do Paraíba do Sul. This gradient is what allows the ten basins to span the full range from near-pristine to severely impaired conditions within a single regulatory jurisdiction and a single monitoring protocol.

The European dataset is the Global River Water Quality Archive (GRQA) v1.4 (Virro et al., 2025), obtained from Zenodo (DOI: 10.5281/zenodo.15335450) under a CC-BY 4.0 licence. GRQA aggregates harmonised measurements from national monitoring programmes across 23 countries: AT, BE, BG, CH, DE, DK, EE, ES, FI, FR, HR, IE, IS, IT, LT, LV, NL, NO, PL, PT, SE, SI, SK, covering 11,638 monitoring stations and 998,410 records (2016–2023). All stations have complete latitude/longitude coordinates.

**Table 1:** European pooled benchmark values (GRQA v1.4, 2016–2022) and corresponding median values for the ten São Paulo basins. All values are empirical medians of the respective distributions. Sample sizes are counts within the shared 2016–2022 analytical window; the Brazilian counts are consequently much smaller than the whole-archive totals, since the CETESB record extends back to 1978

Parameter	Unit	EU benchmark	BR median	Ratio BR/EU	$n_{\textrm{EU}}$	$n_{\textrm{BR}}$
pH	–	7.90	7.10	0.90	121,883	3274
DO	mg L$^{-1}$	9.70	6.00	0.62	109,217	3271
BOD$_5$	mg L$^{-1}$	2.00	3.00	1.50	133,618	2636
TP	mg L$^{-1}$	0.060	0.130	2.15	252,433	3271
Turbidity	NTU	7.00	18.00	2.57	78,435	3274
Temperature	°C	11.50	23.00	2.00	134,241	3268
NO$_3^-$	mg L$^{-1}$	0.768	0.800	1.04	168,209	3269

A shared analytical window of 2016–2022 (six complete years) was selected to maximise temporal overlap while remaining within the GRQA coverage period. Seven physicochemical parameters are common to both datasets with compatible analytical methods: pH (dimensionless), dissolved oxygen (DO, mg L$^{-1}$), biochemical oxygen demand after five days at 20°C (BOD$_5$, mg L$^{-1}$), total phosphorus (TP, mg L$^{-1}$), turbidity (NTU), water temperature (T, °C), and nitrate (NO$_3^-$, mg L$^{-1}$). Brazilian parameter names were mapped to canonical identifiers: *Oxigênio Dissolvido* → DO; *DBO (5, 20)* → BOD$_5$; *Fósforo Total* → TP; *Turbidez* → turbidity; *Temperatura da Água* → temperature; *Nitrogênio-Nitrato* → NO$_3^-$. Values reported in legacy units were converted to SI: temperature in °C, nitrate as NO$_3^-$ (not NO$_3^-$-N). The European benchmark for each parameter is defined as the pooled median of all GRQA measurements in the 2016–2022 window (Table 1).

**Fig. 1:**
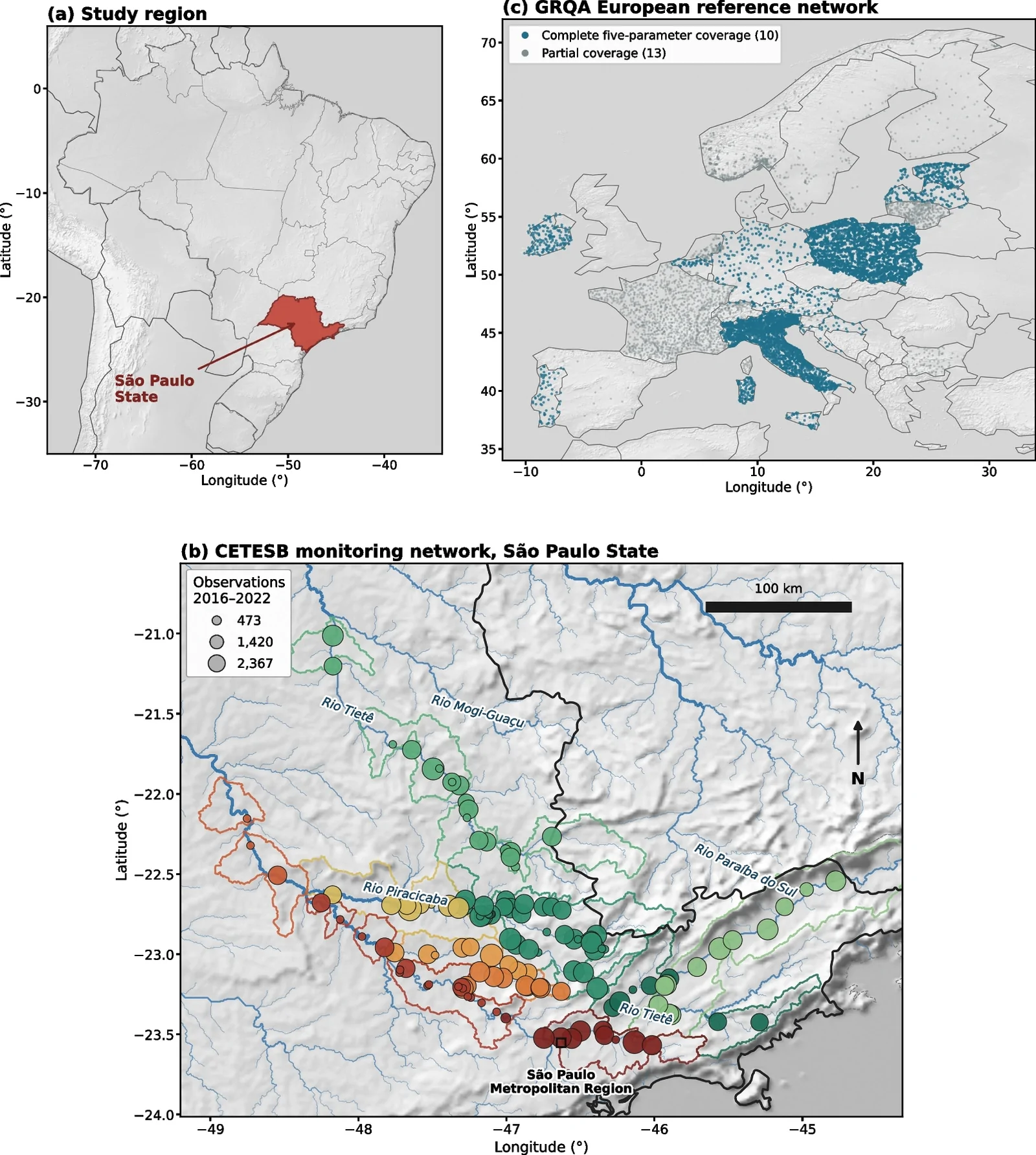
Study area and monitoring networks. **a** Location of São Paulo State (red) within southeastern South America, over shaded relief. **b** CETESB monitoring network in São Paulo State over shaded relief, with the drainage network shown for streams of Strahler order ≥3 and line width scaled by stream order. State boundaries are dashed and the São Paulo boundary is solid. Coloured outlines delineate the ten monitored basins; because no public boundary file is distributed with the CETESB data, these envelopes were derived from the hydrology by assigning each HydroBASINS level-8 sub-basin (Lehner & Grill, 2013) to the study basin containing the majority of its monitoring stations and dissolving the result, and are therefore approximate. Stations (n=141) are coloured by river basin, ordered from the clean-reference group to the most impaired, and symbol area is proportional to the number of valid observations contributed in the 2016–2022 analytical window. The elevation gradient from the Serra do Mar and Serra da Mantiqueira escarpments in the southeast to the Paraná sedimentary basin in the northwest is visible in the relief, as is the concentration of stations in the densely urbanised upper Tietê around the São Paulo Metropolitan Region. **c** GRQA v1.4 European reference network within the mapped extent ($n = 11{,}576$ of 11,638 stations; the remainder lie in overseas territories outside the frame but are retained in all analyses). Stations in the ten countries with complete five-parameter coverage, which form the ranking subset, are distinguished from those in the thirteen countries with partial coverage, which contribute to the Wasserstein and Mann–Whitney analyses only. Basemap: Natural Earth (public domain); drainage network: HydroRIVERS v1.0 (Linke et al., 2019)

Results reported by CETESB as below the analytical detection limit are flagged in the source data and were used at the reported limit value. Within the analytical window, this affects 41.2% of BOD$_5$ determinations, 14.3% of nitrate, 7.6% of total phosphorus, and less than 3% of the remaining parameters. Simple substitution at the detection limit is known to bias summary statistics upward for heavily censored variables (Helsel et al., 2020); the direction of that bias is conservative with respect to the comparisons made here, since it overstates rather than understates the divergence between the Brazilian and European distributions. The implications are discussed further in the “Discussion” section. The spatial distribution of both monitoring networks is shown in Fig. 1.

The two analytical families in this study place different demands on data coverage. The Wasserstein-1 and Mann–Whitney analyses operate on the full pooled empirical distributions and therefore use every available measurement from all ten Brazilian basins and all 23 European countries. The entity-level TOPSIS and PCA/clustering analyses, by contrast, require a complete country-level median for each of the five ranking parameters (pH, DO, BOD$_5$, TP, NO$_3^-$). Ten of the 23 European countries satisfy this requirement over 2016–2022 (AT, BE, DE, EE, HR, IE, IT, LV, PL, PT); the remaining thirteen lack a within-window median for at least one ranking parameter — most commonly BOD$_5$, which is not reported in a harmonisable form for several Nordic and Iberian networks in GRQA v1.4 (Virro et al., 2025) — and are therefore excluded from the decision matrix rather than imputed, to avoid introducing imputation artefacts into the rankings. The TOPSIS and PCA results below thus describe a 20-entity comparison set, while the distributional (Wasserstein and Mann-Whitney) results retain the full data from all entities.

### Wasserstein-1 distributional distance

For each combination of Brazilian basin *b* and parameter *j*, the Wasserstein-1 distance between the empirical distribution of measurements $\mu _{bj}$ and the pooled European empi-rical distribution $\nu _j$ was computed following Eq. (2), implemented via sorted-array integration (equivalent to scipy.stats.wasserstein_distance in Python 3.12, which computes the $L^1$-Wasserstein distance between two one-dimensional empirical distributions).

A composite normalised $W_1$ score was computed for each basin across the five policy-relevant parameters (DO, BOD$_5$, TP, turbidity, NO$_3^-$; temperature excluded as it reflects climatic differences rather than anthropogenic impairment; pH excluded due to near-negligible variation across entities):

3 $$\begin{aligned} \overline{W}_1(b) = \frac{1}{|\mathcal {P}|} \sum _{j \in \mathcal {P}} \frac{W_1(\mu _{bj},\,\nu _j)}{R_j^{(95-5)}} \end{aligned}$$

where $\mathcal {P} = \{\mathrm {DO, BOD_5, TP, turbidity, NO_3^-}\}$ and $R_j^{(95-5)}$ is the 95th–5th percentile range of the pooled Brazil–Europe distribution for parameter *j*, normalising distances to a common robust scale that is insensitive to extreme outliers.

### Mann–Whitney U test and Cliff’s δ Effect Size

For each parameter *j*, a two-sided Mann–Whitney U test was applied to compare the pooled Brazilian distribution (all ten basins combined, 2016–2022) against the pooled European distribution. The Cliff’s δ non-parametric effect size (Cliff, 1993) was computed as:

4 $$\begin{aligned} \delta = \frac{\#(x_i > y_j) - \#(x_i < y_j)}{n_1 \cdot n_2} \end{aligned}$$

where $x_i$ and $y_j$ are Brazilian and European measurements respectively, and $n_1$, $n_2$ are the respective sample sizes. Cliff’s $\delta \in [-1, +1]$, with conventional thresholds: negligible (|δ|<0.147), small ($0.147 \le |\delta | < 0.330$), medium ($0.330 \le |\delta | < 0.474$), and large ($|\delta | \ge 0.474$). Because the product $n_1 n_2$ exceeds $10^8$ for several parameters, δ was evaluated by sorted-array rank counting on random subsamples of at most 20,000 observations per group under a fixed seed; repeated draws vary the reported values by less than 0.015, which does not alter any effect-size classification.

### Shannon-entropy-weighted TOPSIS

A decision matrix $\textbf{D} \in \mathbb {R}^{20 \times 5}$ was constructed using the median values of five parameters (pH, DO, BOD$_5$, TP, NO$_3^-$) for each of the 20 entities with complete parameter coverage (10 Brazilian basins and 10 European countries) over 2016–2022. Temperature was excluded to avoid conflating climatic with anthropogenic differences. The two parameters with coverage below 70% across entities (turbidity, after excluding missing European entries) were excluded from the decision matrix to prevent imputation artefacts from distorting rankings.

Shannon entropy weights (Shannon, 1948) were computed column-wise:

5 $$\begin{aligned} e_j = -\frac{1}{\ln n} \sum _{i=1}^{n} p_{ij} \ln p_{ij}, \quad p_{ij} = \frac{d_{ij}}{\displaystyle \sum _{i=1}^{n} d_{ij}} \end{aligned}$$

6 $$\begin{aligned} w_j = \frac{1 - e_j}{\displaystyle \sum _{k=1}^{m}(1 - e_k)} \end{aligned}$$

where n=20 entities, m=5 parameters, and $d_{ij}$ is the raw decision matrix entry. The resulting weights reflect informational discriminating power: parameters with greater cross-entity variability receive higher weights.

TOPSIS was applied to the normalised weighted decision matrix following Hwang and Yoon (1981). DO was treated as a benefit criterion (higher is better); pH, BOD$_5$, TP, and NO$_3^-$ were treated as cost criteria (lower is better) for the purpose of identifying the positive ideal solution. The TOPSIS score $C_i^* \in [0,1]$ represents the relative closeness to the ideal:

7 $$\begin{aligned} C_i^* = \frac{d_i^-}{d_i^+ + d_i^-} \end{aligned}$$

where $d_i^+$ and $d_i^-$ are the Euclidean distances of entity *i* from the positive and negative ideal solutions in the weighted normalised decision space, respectively. Entities with $C_i^* \rightarrow 1$ are closest to the positive ideal.

### Linear programming convergence model

For each Brazilian basin *b* and policy parameter $j \in \mathcal {P}$, the convergence gap $g_{bj}$ is defined as:

8 $$\begin{aligned} g_{bj} = {\left\{ \begin{array}{ll} \max \!\bigl (0,\; \theta _j^* - \mu _{bj}\bigr ) & \text {if parameter } j \text { is a benefit (DO)} \\ \max \!\bigl (0,\; \mu _{bj} - \theta _j^*\bigr ) & \text {if parameter } j \text { is a cost} \end{array}\right. } \end{aligned}$$

where $\mu _{bj}$ is the basin median (2016–2022) and $\theta _j^*$ is the European pooled benchmark.

**Table 2:** Median values of seven physicochemical parameters for each São Paulo basin (2016–2022 window). Values exceeding the European pooled benchmark ($\theta _j^*$) are marked †; values more than twice the benchmark are marked ‡. DO values below the CONAMA Class 2 minimum (5 mg L$^{-1}$) are marked ∗. Temperature is not flagged against the European benchmark, as the difference reflects the tropical versus temperate climate rather than anthropogenic impairment

Basin	pH	DO	BOD$_5$	TP	Turb.	Temp.	NO$_3^-$
	–	mg/L	mg/L	mg/L	NTU	°C	mg/L
EU benchmark	7.90	9.70	2.00	0.060	7.00	11.5	0.768
Afluentes-PSB	7.00	7.10	3.0†	0.021	7.0	22.0	0.200
Cantareira	7.10	6.80	3.0†	0.100†	13.0†	22.6	0.800†
Mogi-Guaçu	7.04	6.55	2.0	0.120†	24.0‡	23.7	0.900†
Paraíba do Sul	6.80	5.20∗	3.0†	0.095†	19.0‡	23.0	0.630
Tietê-Baixo	7.11	4.90∗	3.0†	0.110†	5.1	25.6	3.32‡
Piracicaba	7.00	5.10∗	4.0†	0.300‡	15.0†	24.3	1.200†
Capivari	7.35	5.20∗	5.0†	0.400‡	18.0†	22.3	1.850†
Jundiaí	7.30	6.60	9.0‡	0.400‡	20.0‡	22.6	2.000‡
Tietê-Médio	7.37	3.50∗	9.0‡	1.000‡	16.0†	24.8	1.140†
Tietê-Alto	7.08	0.94∗	13.4‡	0.850‡	28.0‡	22.1	0.200

The minimum-cost convergence LP is formulated as:

9 $$\begin{aligned} \min _{\textbf{r}} \quad \textbf{w}^\top \textbf{r} \end{aligned}$$

10 $$\begin{aligned} \text {s.t.} \quad r_{bj} \ge g_{bj} \quad \forall \, b,j \end{aligned}$$

11 $$\begin{aligned} 0 \le r_{bj} \le r_{j}^{\max } \cdot T \quad \forall \, b,j \end{aligned}$$

where $r_{bj}$ is the required improvement in parameter *j* for basin *b*, $\textbf{w}$ is the vector of LP cost weights (derived as the normalised inverse of the feasibility rate $r_j^{\max }$), and *T* is the planning horizon in years. The bound $r_j^{\max }$ is estimated empirically from the CETESB record as the 90th percentile of the *positive* year-on-year changes in the state-wide annual median of parameter *j*, computed over the full observational record (1978–2026) in the improving direction for that parameter (increasing for DO, decreasing for the cost parameters). It therefore represents the fastest state-level improvement historically sustained over a single year. Because gains in individual basins partially offset one another when medians are aggregated to the state level, this bound is more conservative than a basin-specific maximum would be. This anchors the LP to demonstrated real-world feasibility rather than engineering hypotheticals. A sensitivity analysis replacing the 90th with the 75th percentile is reported in the “Discussion” section.

The critical-path convergence time for basin *b* is:

12 $$\begin{aligned} T_b^* = \max _{j \in \mathcal {P}}\; \frac{g_{bj}}{r_j^{\max }} \end{aligned}$$

representing the number of years required at maximum improvement rates until the slowest parameter reaches the European benchmark. Because $r_j^{\max }$ varies by an order of magnitude across parameters (turbidity: 12.1 NTU yr$^{-1}$; TP: 0.074 mg L$^{-1}$ yr$^{-1}$), the binding constraint is determined by the gap-to-feasibility ratio, not the absolute gap magnitude.

### PCA and Ward hierarchical cluster analysis

PCA was applied to the *z*-score-standardised version of the 20×5 decision matrix to identify orthogonal dimensions of variation across entities. Ward linkage hierarchical cluster analysis (Ward, 1963) was performed on the standardised matrix (Euclidean distance), and the dendrogram was cut at k=4 clusters based on the elbow criterion applied to the within-cluster sum of squares.

All analyses were implemented in Python 3.12 using pandas 2.2, numpy 1.26, scipy 1.13, and scikit-learn 1.4.

## Results

### Distributional characterisation: basin medians and European benchmark

Table 1 summarises the seven-parameter comparison of pooled Brazilian and European median values for 2016–2022. The most extreme absolute divergence is observed for turbidity (Brazilian median 18.0 NTU versus European 7.0 NTU; ratio = 2.57) and total phosphorus (0.130 vs. 0.060 mg L$^{-1}$; ratio = 2.15). Dissolved oxygen is 38% below the European reference (6.00 vs. 9.70 mg L$^{-1}$). Remarkably, nitrate is the sole parameter where Brazil and Europe are essentially at parity (0.800 vs. 0.768 mg L$^{-1}$; ratio = 1.04), a finding with nuanced management implications discussed in the “Discussion” section.

**Fig. 2:**
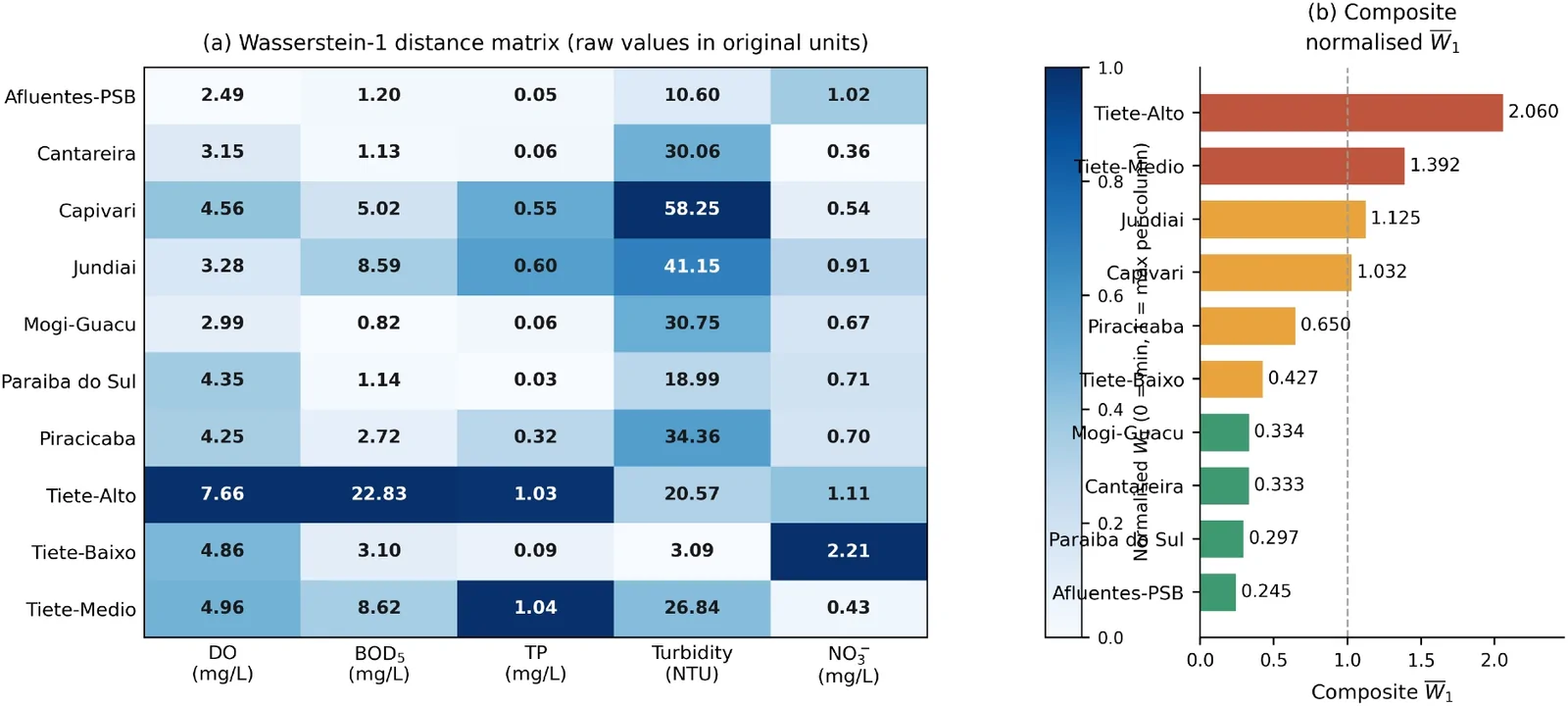
Wasserstein-1 distance matrix (basins × parameters). Colour scale: white = minimum $W_1$ (closest to European distribution); dark blue = maximum $W_1$. Numbers in cells are raw $W_1$ distances in the original units of each parameter. The rightmost column shows the composite normalised score $\overline{W}_1$ (Eq. 3)

**Table 3:** Wasserstein-1 distances between each São Paulo basin and the pooled European distribution (GRQA v1.4, 2016–2022), and composite normalised score $\overline{W}_1$. Smaller values indicate distributional proximity to the European reference

Basin	$W_1$(DO)	$W_1$(BOD)	$W_1$(TP)	$W_1$(Turb)	$W_1$(NO$_3$)	$\overline{W}_1$
Afluentes-PSB	2.486	1.200	0.052	10.60	1.023	0.245
Paraíba do Sul	4.350	1.135	0.029	18.99	0.707	0.297
Cantareira	3.153	1.130	0.062	30.06	0.358	0.333
Mogi-Guaçu	2.991	0.821	0.063	30.75	0.665	0.334
Tietê-Baixo	4.863	3.104	0.093	3.09	2.208	0.427
Piracicaba	4.246	2.716	0.320	34.36	0.697	0.650
Capivari	4.564	5.021	0.545	58.25	0.539	1.032
Jundiaí	3.276	8.585	0.597	41.15	0.907	1.125
Tietê-Médio	4.958	8.623	1.038	26.84	0.427	1.392
Tietê-Alto	7.661	22.828	1.026	20.57	1.107	2.060

Basin-level medians reveal the full range of this heterogeneity (Table 2). Dissolved oxygen ranges from 0.94 mg L$^{-1}$ (Tietê-Alto, severe chronic anoxia) to 7.10 mg L$^{-1}$ (Afluentes-PSB, above the CONAMA Class 2 threshold of 5 mg L$^{-1}$ (CONAMA, 2005)). BOD$_5$ spans from 2.0 mg L$^{-1}$ (Mogi-Guaçu, within European range) to 13.4 mg L$^{-1}$ (Tietê-Alto, 6.7× the European benchmark). TP in the Tietê-Médio (1.000 mg L$^{-1}$) exceeds the European benchmark by a factor of 16.6.

**Table 4:** Mann–Whitney U test and Cliff’s δ effect size for distributional comparison of pooled Brazilian (all ten basins, $n_{\textrm{BR}}$ = 2636–3274) versus European GRQA v1.4 ($n_{\textrm{EU}}$ = 78,435–252,433) measurements, both restricted to the shared 2016–2022 window. Effect size thresholds: negligible |δ|<0.147; small 0.147–0.330; medium 0.330–0.474; large >0.474

Parameter	BR median	EU median	*p*-value	Cliff’s δ	Effect	Direction
pH	7.10	7.90	<0.001	-0.717	large	BR < EU
DO	6.00	9.70	<0.001	-0.768	large	BR < EU
BOD$_5$	3.00	2.00	<0.001	+0.571	large	BR > EU
TP	0.130	0.060	<0.001	+0.439	medium	BR > EU
Turbidity	18.00	7.00	<0.001	+0.510	large	BR > EU
Temperature	23.00	11.50	<0.001	+0.858	large	BR > EU
NO$_3^-$	0.800	0.768	0.354	-0.010	negligible	n.s.

**Table 5:** Shannon entropy weights for the five TOPSIS criteria (20-entity decision matrix, 2016–2022)

Parameter	Weight $w_j$	Interpretation
TP	0.472	Highest cross-entity variability
BOD$_5$	0.239	Second most discriminating
NO$_3^-$	0.181	Third most discriminating
DO	0.107	Moderate discrimination
pH	0.001	Near-uniform across entities

### Wasserstein-1 distributional distance analysis

Figure 2 presents the full $W_1$ distance matrix across ten basins and seven parameters, and Table 3 presents the composite normalised scores. The Tietê-Alto exhibits the largest $W_1$ distances in all organic and oxygen parameters: $W_1(\textrm{DO}) = 7.661$ mg L$^{-1}$ and $W_1(\mathrm {BOD_5}) = 22.828$ mg L$^{-1}$ — values that reflect not merely a higher median but a fundamentally different distributional regime, with a substantial mass of observations near zero DO and above 20 mg L$^{-1}$ BOD$_5$.

A key observation is that the composite $\overline{W}_1$ of the Tietê-Alto (2.060) is 8.4 times greater than that of Afluentes-PSB (0.245), despite both being São Paulo basins subject to the same regulatory framework. The Cantareira system ($\overline{W}_1 = 0.333$) and Mogi-Guaçu ($\overline{W}_1 = 0.334$) are at virtual parity in distributional proximity to the European reference, and both are within the range expected for a mid-performing European country. Figure 2 shows that the turbidity parameter contributes disproportionately to the absolute $W_1$ distances due to its larger numeric scale; normalisation by the 95–5 percentile range substantially reduces this dominance in the composite score.

### Mann–Whitney U and Cliff’s δ results

Table 4 presents the distributional comparison results for all seven parameters, computed on the shared 2016–2022 window for both the Brazilian and European pooled distributions. Six parameters exhibit statistically significant differences between the Brazilian and European distributions at α=0.01. The largest effect sizes are observed for temperature (δ=+0.858, large; Brazilian values higher, as expected from tropical climate), DO (δ=-0.768, large; Brazilian values stochastically lower), pH (δ=-0.717, large; Brazilian values lower, reflecting different buffering characteristics of tropical soils), and BOD$_5$ (δ=+0.571, large; Brazilian values higher). Nitrate is the sole parameter without significant distributional difference (p=0.354; δ=-0.010, negligible), a finding elaborated in the Discussion.

### TOPSIS ranking of complete-coverage entities

Tables 5 and 6 present the Shannon entropy weights and the full TOPSIS ranking, respectively. The decision matrix comprises the 20 entities with complete five-parameter coverage (all ten São Paulo basins and the ten European countries AT, BE, DE, EE, HR, IE, IT, LV, PL, PT). TP receives by far the highest weight (w=0.472), reflecting its high cross-entity variability spanning more than a thirty-fold range (from 0.030 mg L$^{-1}$ in Austria to 1.000 mg L$^{-1}$ in Tietê-Médio). BOD$_5$ (w=0.239) and NO$_3^-$ (w=0.181) are the second and third most discriminating parameters; DO (w=0.107) and pH (w=0.001) contribute little additional information beyond what TP already encodes.

**Table 6:** Full TOPSIS ranking of the 20 entities with complete five-parameter coverage (10 São Paulo basins and 10 European countries), 2016–2022. Shannon entropy weights in Table 5. Score $C_i^* \in [0,1]$; higher values indicate proximity to the positive ideal solution. Brazilian basins are in **bold**. The 13 European countries lacking a country-level median for at least one of the five ranking parameters (BG, CH, DK, ES, FI, FR, IS, LT, NL, NO, SE, SI, SK) are necessarily excluded from the decision matrix but are retained in the full-distribution Wasserstein and Mann–Whitney analyses

Rank	Entity	$C_i^*$	Type
1	Latvia (LV)	0.956	Europe
2	Austria (AT)	0.937	Europe
3	Croatia (HR)	0.925	Europe
4	Estonia (EE)	0.922	Europe
5	Ireland (IE)	0.917	Europe
6	Italy (IT)	0.905	Europe
7	**Afluentes-PSB**	**0.901**	**Brazil**
8	Portugal (PT)	0.892	Europe
9	Poland (PL)	0.879	Europe
10	**Cantareira**	**0.867**	**Brazil**
11	**Paraíba do Sul**	**0.867**	**Brazil**
12	**Mogi-Guaçu**	**0.864**	**Brazil**
13	Germany (DE)	0.817	Europe
14	**Tietê-Baixo**	**0.754**	**Brazil**
15	**Piracicaba**	**0.708**	**Brazil**
16	Belgium (BE)	0.695	Europe
17	**Capivari**	**0.607**	**Brazil**
18	**Jundiaí**	**0.553**	**Brazil**
19	**Tietê-Alto**	**0.246**	**Brazil**
20	**Tietê-Médio**	**0.194**	**Brazil**

**Fig. 3:**
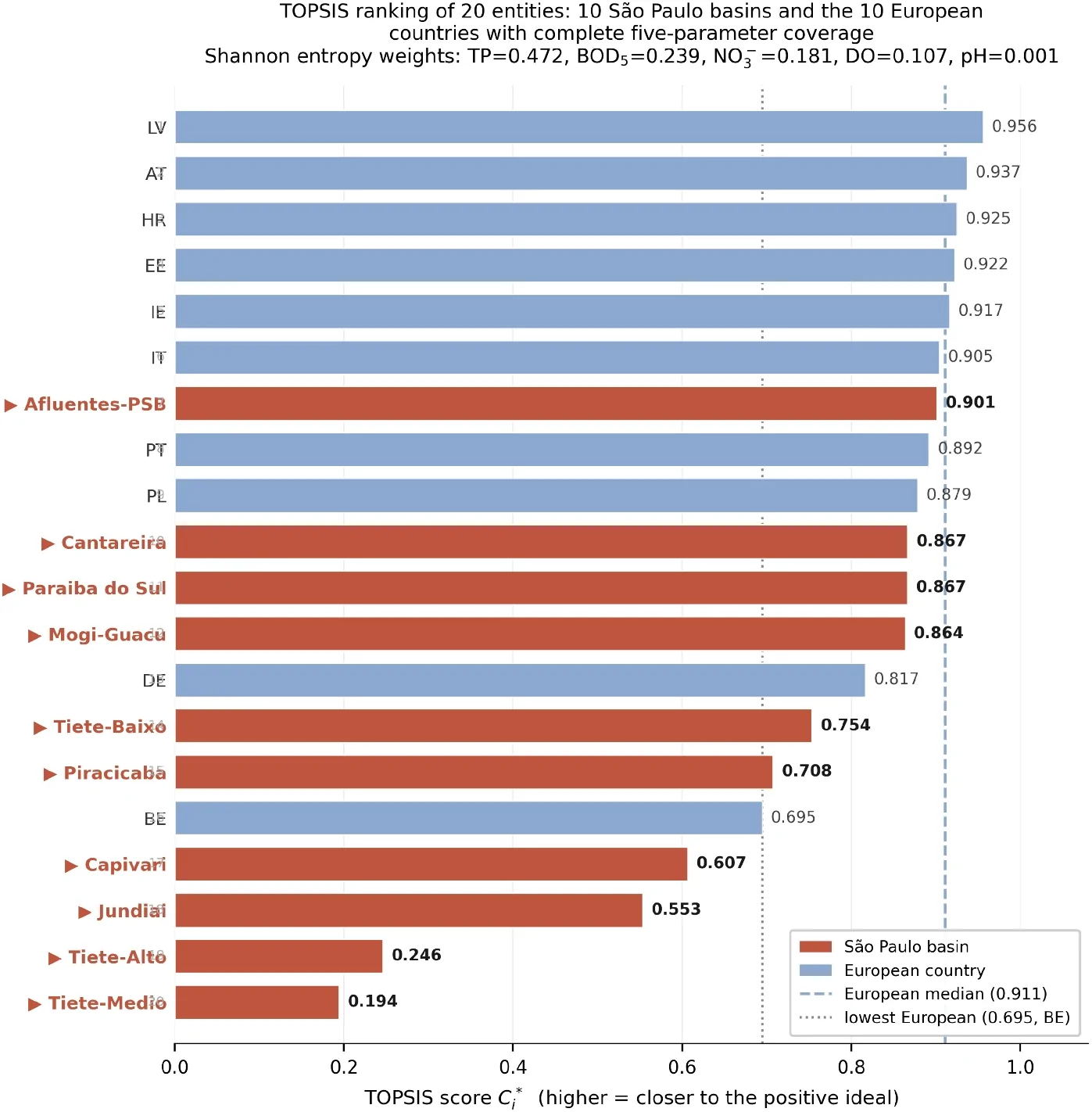
TOPSIS score distribution for the 20 complete-coverage entities. Blue bars: European countries. Orange bars: São Paulo basins. Dotted vertical line: median European score. The bimodal Brazilian distribution — six basins scoring above the lowest-ranked European country and four falling below it, with a pronounced gap between Piracicaba (0.708) and Capivari (0.607) — is a central finding of this study

The TOPSIS results reveal that São Paulo’s river network is bifurcated in quality terms. Four basins (Afluentes-PSB 7th, Cantareira 10th, Paraíba do Sul 11th, Mogi-Guaçu 12th) rank within the European distribution and above major European economies including Germany, Poland, and Belgium; Afluentes-PSB in particular ranks ahead of Portugal, Poland, Germany, and Belgium. In contrast, Tietê-Alto (19th) and Tietê-Médio (20th) rank below all ten complete-coverage European countries, constituting distributional outliers whose quality profiles are unlike any country in the complete-coverage GRQA reference set. The median European TOPSIS score is 0.911; the four clean-reference Brazilian basins cluster immediately below this median, within the range spanned by Germany and Portugal. Figure 3 visualises this bimodal structure.

### Linear programming convergence analysis

Table 7 presents the convergence gap $g_{bj}$ for each basin-parameter combination. The historically observed maximum improvement rates from the CETESB record ($r_j^{\max }$) vary substantially across parameters: turbidity can improve rapidly (12.1 NTU yr$^{-1}$) through sediment control measures, while TP responds slowly (0.074 mg L$^{-1}$ yr$^{-1}$) even under intensive treatment programmes. This asymmetry in feasibility rates is decisive: despite TP carrying the highest LP cost weight ($w_j = 0.669$), dissolved oxygen is the binding rate-limiting constraint for seven of ten basins because the DO gap-to-feasibility ratio ($g_{bj}/r_j^{\max }$) exceeds that of any other parameter. Total phosphorus is the binding constraint for one basin only (Tietê-Médio), and nitrate for two (Jundiaí and Tietê-Baixo).

Afluentes-PSB, whose TP gap is zero (current median of 0.021 mg L$^{-1}$ is already below the European benchmark of 0.060 mg L$^{-1}$), requires 4.4 years of sustained DO improvement at maximum historical rate to converge. The Tietê-Alto requires 14.8 years, bounded by dissolved oxygen: the DO gap of 8.76 mg L$^{-1}$ at 0.59 mg L$^{-1}$ yr$^{-1}$ yields 14.8 years, whereas BOD$_5$ — despite carrying the largest absolute gap in the entire matrix (11.4 mg L$^{-1}$) — resolves in 4.6 years at 2.5 mg L$^{-1}$ yr$^{-1}$. This contrast illustrates the central mechanism of the model: the binding constraint is set by the gap-to-feasibility ratio, not by the magnitude of the gap itself. It also means that even if every other Tietê-Alto parameter reached European levels tomorrow, dissolved oxygen alone would still require nearly fifteen years of sustained improvement at the best annual rate the state has historically achieved.

**Fig. 4:**
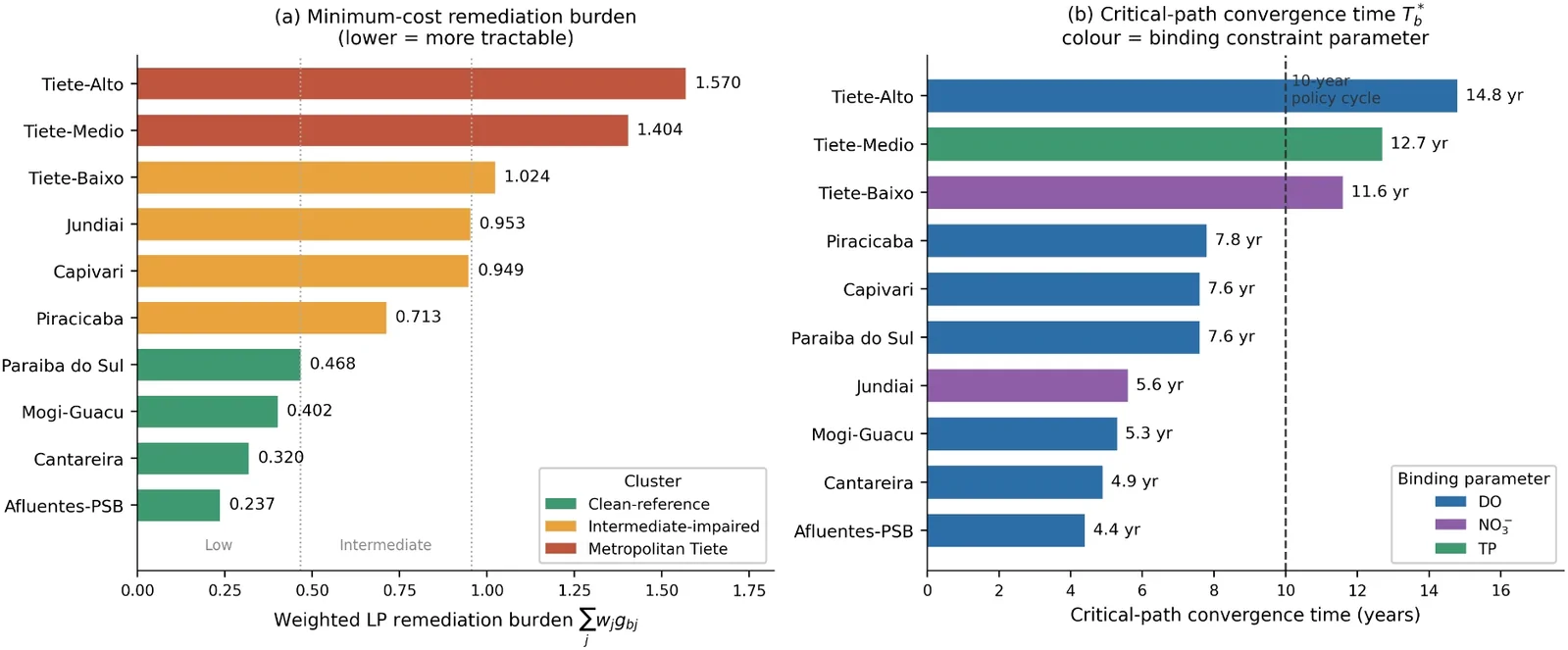
LP convergence planning results. Left: weighted remediation burden $\sum _j w_j g_{bj}$ per basin (lower = more tractable). Right: critical-path convergence time $T_b^*$ per basin with parameter colour-coding of the binding constraint. Three burden tiers are delineated by dashed horizontal lines

Figure 4 presents the parametric efficiency frontier, showing the weighted LP burden as a function of target convergence year. Three distinct burden tiers emerge: low-burden basins (Afluentes-PSB, Cantareira, Mogi-Guaçu, Paraíba do Sul; burden ≤0.47) that are plausibly convergent within a regulatory decade under current trajectory; intermediate-burden basins (Piracicaba, Capivari, Jundiaí, Tietê-Baixo; burden 0.71–1.02) that require sustained multi-parameter intervention; and the Tietê metropolitan arc (Tietê-Médio and Tietê-Alto; burden 1.40–1.57) that requires structural infrastructure transformation beyond any single-parameter remediation.

### PCA and typological clustering

**Fig. 5:**
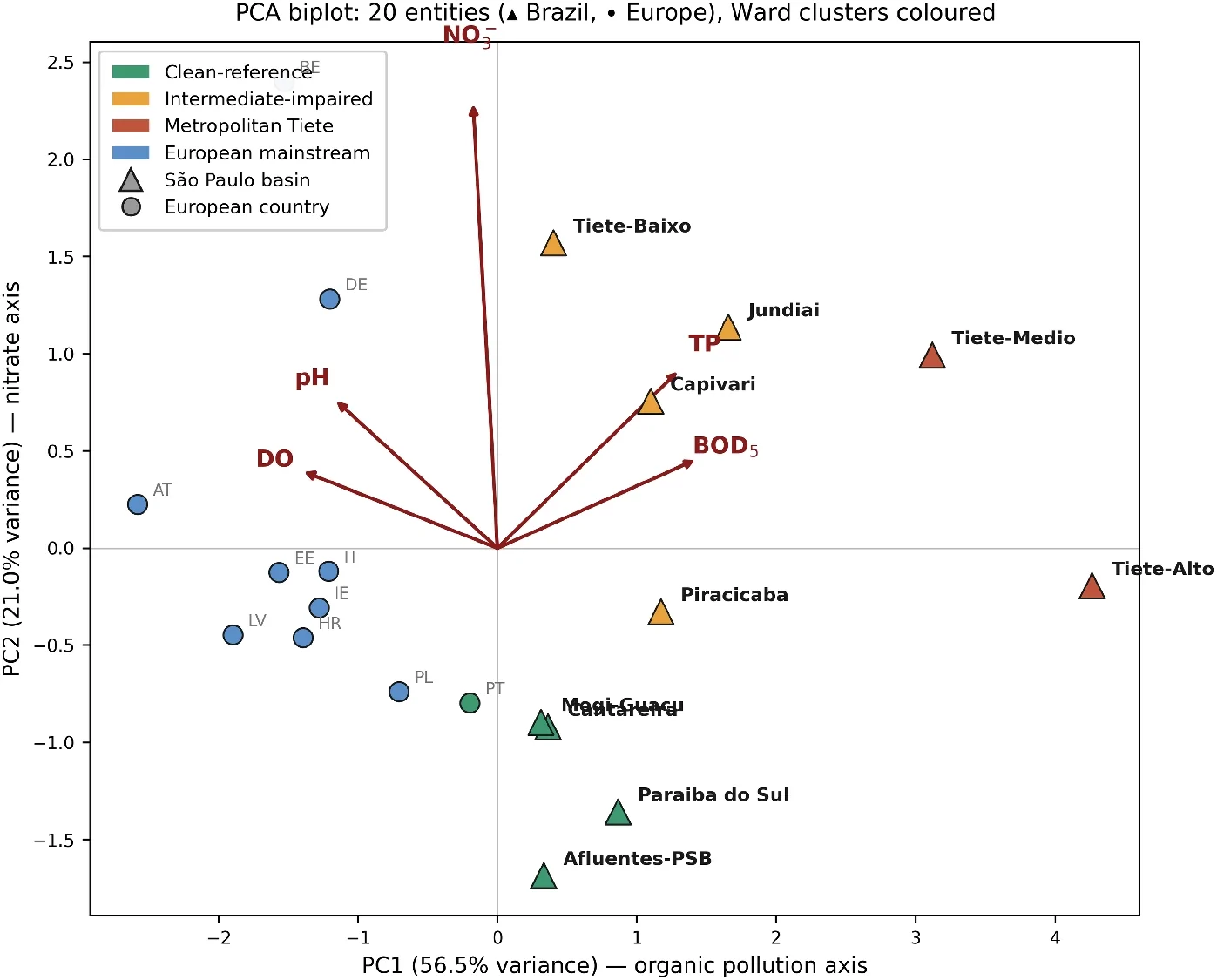
PCA biplot of the 20-entity decision matrix (five parameters, 2016–2022). Ellipses indicate the four Ward clusters. Brazilian basins: filled triangles; European countries: filled circles. Parameter vectors (loadings) shown for PC1 and PC2. PC1 (56.5%): organic pollution axis; PC2 (21.0%): nitrate axis

PCA of the standardised 20×5 decision matrix (pH, DO, BOD$_5$, TP, NO$_3^-$) explains 77.5% of total variance in two components (PC1: 56.5%; PC2: 21.0%; Fig. 5). PC1 is a pollution axis: positive loadings on BOD$_5$ (+0.537) and TP (+0.490) oppose negative loadings on DO (-0.526) and pH (-0.437), consistently with the co-occurrence of organic enrichment, nutrient loading, and deoxygenation in impaired systems. PC2 is a nitrate axis dominated by a strong positive loading on NO$_3^-$ (+0.865), separating high-nitrate systems (the agricultural Piracicaba–Capivari–Jundiaí catchments and several Central European countries) from low-nitrate systems (Afluentes-PSB, Tietê-Alto; low PC2 scores).

**Table 7:** Convergence gap $g_{bj}$ (required improvement to reach European benchmark $\theta _j^*$), maximum feasible improvement rate $r_j^{\max }$ (90th percentile of the positive year-on-year changes in the state-wide annual median, CETESB 1978–2026), LP cost weight $w_j$ (normalised inverse of $r_j^{\max }$), and critical-path convergence time $T_b^*$ (years to reach European benchmark on the slowest parameter)

Basin	*g*(DO)	*g*(BOD)	*g*(TP)	*g*(Turb)	*g*(NO$_3$)	$T_b^*$ (yr)
	mg/L	mg/L	mg/L	NTU	mg/L	
$\theta _j^*$	9.70	2.00	0.060	7.00	0.768	—
$r_j^{\max }$	0.59	2.50	0.074	12.1	0.220	—
LP cost weight $w_j$	0.084	0.020	0.669	0.004	0.224	—
Afluentes-PSB	2.60	1.00	0.000	0.0	0.000	**4.4**
Cantareira	2.90	1.00	0.040	6.0	0.032	**4.9**
Mogi-Guaçu	3.16	0.00	0.060	17.0	0.132	**5.4**
Jundiaí	3.10	7.00	0.340	13.0	1.232	**5.6**
Capivari	4.50	3.00	0.340	11.0	1.082	**7.6**
Paraíba do Sul	4.50	1.00	0.035	12.0	0.000	**7.6**
Piracicaba	4.60	2.00	0.240	8.0	0.432	**7.8**
Tietê-Baixo	4.80	1.00	0.050	0.0	2.547	**11.6**
Tietê-Médio	6.20	7.00	0.940	9.0	0.372	**12.7**
Tietê-Alto	8.76	11.40	0.790	21.0	0.000	**14.8**

**Fig. 6:**
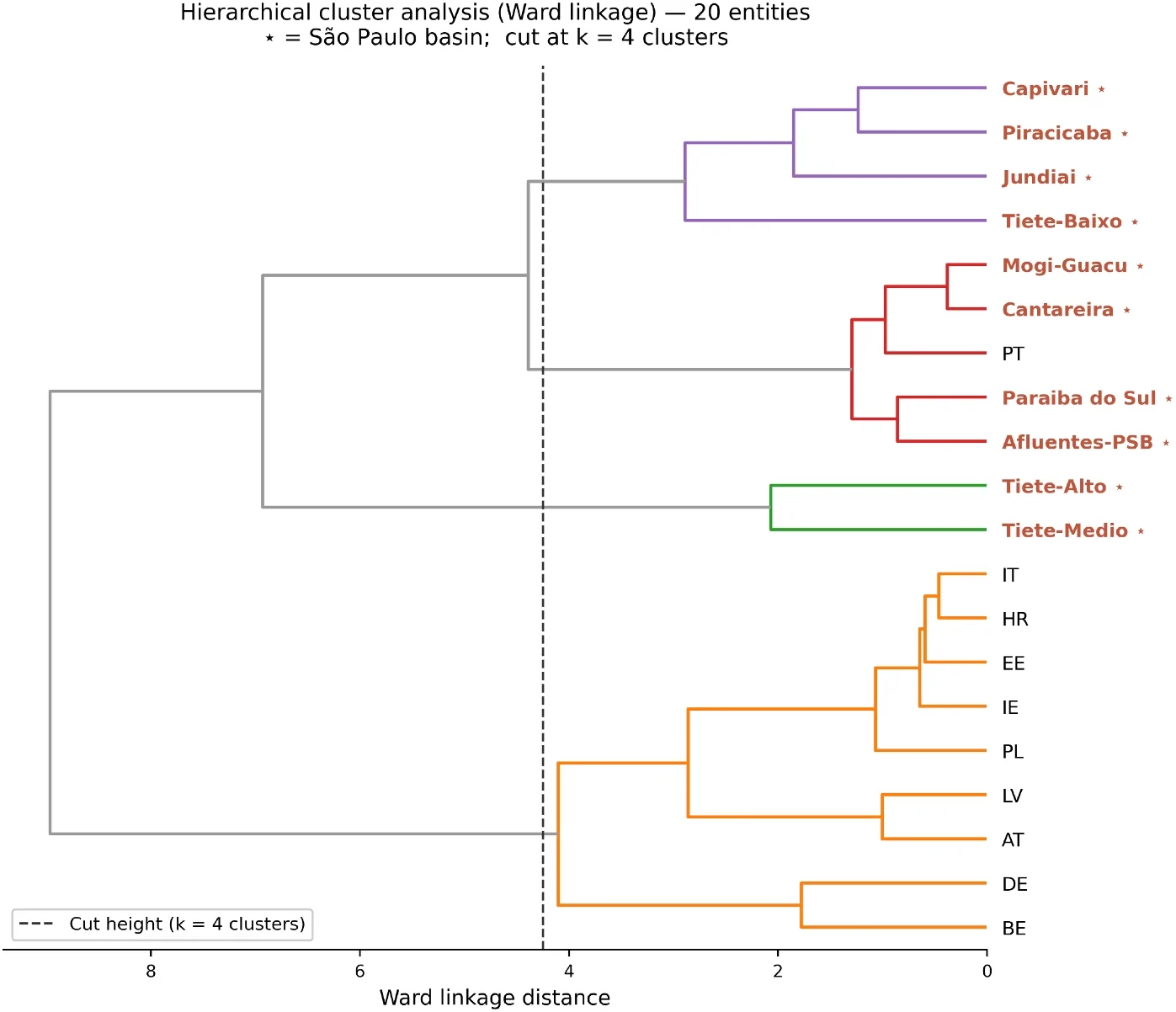
Ward linkage dendrogram for all 20 entities. Coloured branches indicate the four clusters. Horizontal dashed line: dendrogram cut height. Brazilian basins are annotated with $^{\star }$

Ward HCA identifies four clusters (Fig. 6):

- **Cluster 1 — European mainstream**: nine of the ten complete-coverage European countries (AT, BE, DE, EE, HR, IE, IT, LV, and PL); the tenth, Portugal, clusters instead with the clean-reference group (Cluster 3). Predominantly negative PC1 (generally clean to moderately impaired) and variable PC2 (reflecting the diversity of nitrate loading across Central and Western Europe). Germany and Belgium sit at the lower end of European TOPSIS scores (0.817 and 0.695 respectively), consistent with documented diffuse agricultural nitrate pressure.
- **Cluster 2 — Metropolitan Tietê arc**: Tietê-Alto and Tietê-Médio. Extreme positive PC1 scores, reflecting severe chronic impairment by organic loading and deoxygenation. These basins are statistical outliers from the entity set; no European country in the comparison occupies this quadrant.
- **Cluster 3 — Clean-reference systems**: the four Brazilian basins Afluentes-PSB, Cantareira, Mogi-Guaçu, and Paraíba do Sul, together with Portugal. Negative PC1 scores confirm low organic loading, low nutrients, and moderate alkalinity consistent with headwater or well-forested catchments. This constitutes the study’s most actionable positive finding: four São Paulo basins exhibit distributional profiles that place them within the European reference range rather than as outliers, across the five physicochemical parameters analysed, while acknowledging that broader ecological indicators are not captured here.
- **Cluster 4 — Intermediate-impaired**: Capivari, Jundiaí, Piracicaba, and Tietê-Baixo. Positive PC1 (elevated BOD$_5$ and TP, reduced DO) and elevated PC2 (high NO$_3^-$), corresponding to basins receiving mixed agricultural, industrial, and urban discharges at intermediate loading rates.

## Discussion

### The São Paulo quality bifurcation and its governance implications

The central finding of this study is a structural bifurcation in São Paulo’s river quality that is invisible to aggregate reporting but clearly exposed by distributional analysis. Four headwater and tributary basins — Afluentes-PSB (ranked 7th of 20), Cantareira (10th), Paraíba do Sul (11th), and Mogi-Guaçu (12th) — are embedded within the European quality distribution and form a typological cluster with Portugal at the clean-reference end of the ordination. Two metropolitan Tietê sub-basins (ranked 19th and 20th) are quality outliers with no equivalent among the ten complete-coverage European countries. This bifurcation has a clear structural explanation: the Cantareira, Mogi-Guaçu, and Afluentes-PSB systems drain predominantly forested or low-intensity agricultural catchments with mature wastewater treatment coverage, while the Alto and Médio Tietê receive the cumulative discharge of the São Paulo Metropolitan Region (∼22 million inhabitants), where treatment deficits remain structurally entrenched.

The LP analysis adds a temporal dimension to this bifurcation. The four “European-range” basins face critical-path convergence times of 4.4–7.8 years under sustained maximum improvement rates, suggesting that regulatory compliance at the European standard is not merely aspirational but potentially achievable within a single infrastructure investment cycle, provided that dissolved oxygen recovery — the binding LP constraint for seven of the ten basins — and targeted phosphorus reduction are prioritised. In contrast, the Alto Tietê’s critical-path time of 14.8 years (bounded by dissolved oxygen, given its near-zero baseline median of 0.94 mg L$^{-1}$) implies that even under historically unprecedented improvement rates, convergence to the European benchmark is a generational rather than a political-cycle target. This distinction is managerially significant: it argues for differentiated governance strategies rather than basin-uniform targets.

### The nitrate paradox and agricultural nutrient loading

The finding that nitrate is the sole parameter without a significant distributional difference between Brazilian and European rivers (p=0.354) warrants careful interpretation. Brazilian nitrate concentrations (median 0.800 mg L$^{-1}$) are at parity with the European median (0.768 mg L$^{-1}$), but this apparent convergence may mask divergent underlying processes. The interpretation we offer here is a plausible reading of the monitoring record rather than a demonstrated causal account, and we set it out as such. European nitrate concentrations are widely understood to reflect decades of agricultural intensification followed by regulatory pressure from the Nitrates Directive (91/676/EEC) and the WFD, so that the observed European distribution plausibly combines loading and remediation signals. On the Brazilian side, the basins with the highest nitrate medians — Piracicaba, Capivari, and Jundiaí, at 1.2–2.0 mg L$^{-1}$ — are also those whose catchments (UGRHI 5 and UGRHI 10) carry the most intensive sugarcane, citrus, and soybean cultivation, and Brazil has no regulatory instrument equivalent to the Nitrates Directive. This co-occurrence is suggestive, but the monitoring data alone cannot establish that agricultural expansion caused the observed concentrations, nor can they isolate the contribution of governance from that of land use, hydrology, or population growth.

**Table 8:** Sensitivity of the TOPSIS ranking to the weighting scheme. Weights are given for each of the five ranking criteria; rank agreement with the entropy-weighted ranking is summarised by Kendall’s τ and Spearman’s ρ over all 20 entities

Scheme	pH	DO	BOD$_5$	TP	NO$_3^-$	τ	ρ
Entropy (used here)	0.001	0.107	0.239	0.472	0.181	—	—
Equal weights	0.200	0.200	0.200	0.200	0.200	0.905	0.979
CRITIC	0.329	0.171	0.144	0.145	0.211	0.863	0.964
Standard deviation	0.045	0.490	0.343	0.029	0.093	0.684	0.795

The trend data from the broader CETESB record are consistent with, though not confirmatory of, this reading: nitrate in the Jundiaí basin increased by more than 800% between the early 1990s and the period of this study (Pimentel Tavares et al., 2026). Testing the interpretation properly would require catchment-scale land-use, fertiliser-application, and point-source loading data that lie outside the scope of a monitoring-record analysis, and we identify this as a specific target for future work rather than a gap we can close here. Subject to that caveat, the present distributional parity is most plausibly read as a transient coincidence of trajectories — Brazilian nutrient loading rising toward European levels rather than being held there by governance — and the basin-level analysis reveals the heterogeneity masked at the aggregate level: the agricultural Jundiaí basin has a median NO$_3^-$ of 2.00 mg L$^{-1}$, more than 2.5 times the European median (0.768 mg L$^{-1}$), whereas the headwater Afluentes-PSB basin sits at 0.200 mg L$^{-1}$, well below it. The aggregate parity between Brazil and Europe (Mann–Whitney p=0.354) therefore conceals a strong internal gradient, from near-pristine headwaters to agriculturally enriched sub-basins already exceeding European concentrations.

### Methodological contributions and transferability

The four-method framework developed here has three features that make it generalisable to any country maintaining multi-decadal monitoring records alongside an international reference dataset. First, Wasserstein-1 distance is parameter-agnostic, scale-invariant (via normalisation), and does not require normality or any distributional assumption; it can be applied to any continuous monitoring variable with an empirical distribution. To our knowledge, this is the first application of Wasserstein distributional distance as a cross-continental benchmarking metric for river water quality monitoring networks — a methodological contribution that distinguishes this framework from existing index-based or median-based comparative approaches (Jung et al., 2020; Simian et al., 2025). Second, the LP convergence model’s key innovation — anchoring feasibility constraints to historically observed improvement rates — removes the need for external cost databases that are often unavailable in data-sparse contexts; the monitoring record itself provides the feasibility information. Third, the TOPSIS ranking produces an entity-level score that integrates across parameters in a theoretically principled way, enabling direct comparison of basins and countries on a common scale, consistent with the multi-criteria evaluation approach advocated for water resources management by Tsakiris et al. (2015) and Ward (2025).

To aid practical interpretation of the Wasserstein-1 metric for environmental managers, we note that a $W_1$ value of 1.0 mg L$^{-1}$ for dissolved oxygen, for example, represents the average absolute displacement in mg L$^{-1}$ required across the entire empirical distribution to transform the Brazilian basin’s DO distribution into the European reference distribution. For Tietê-Alto, where $W_1(\textrm{DO}) = 7.661$ mg L$^{-1}$, this means that the cumulative distributional displacement across all monitoring records averages nearly 7.7 mg L$^{-1}$ per observation — corresponding to the difference between near-anoxic conditions (median 0.94 mg L$^{-1}$) and the entire European DO distribution, not merely the median. The composite $\overline{W}_1$ score normalises these values by the 95th–5th percentile range of the pooled distribution, making them directly comparable across parameters with different units and scales.

The Shannon entropy weighting procedure assigns total phosphorus a weight of $w_j = 0.472$, substantially higher than other parameters. This reflects an empirical property of the joint dataset: TP spans a more than thirty-fold range across the 20 entities (from 0.030 mg L$^{-1}$ in Austria to 1.000 mg L$^{-1}$ in Tietê-Médio), generating high informational discriminating power. This is not an analyst choice but an objective consequence of entropy weighting, which assigns influence in proportion to cross-entity variability. However, we acknowledge that this concentration of weight in a single parameter makes TOPSIS rankings sensitive to TP measurement accuracy, and that the dominance of TP should be interpreted alongside the full parameter-specific Wasserstein analysis rather than in isolation.

To test whether the concentration of weight in total phosphorus determines the ranking, TOPSIS was recomputed under three alternative weighting schemes: equal weights, CRITIC (Diakoulaki et al., 1995), and standard-deviation weights (Table 8). Rank agreement with the entropy-weighted ranking is high (Kendall’s τ=0.905 for equal weights, 0.863 for CRITIC, and 0.684 for standard-deviation weights; Spearman’s ρ=0.979, 0.964, and 0.795 respectively). The CRITIC scheme provides the most demanding test, since it redistributes weight away from total phosphorus (from 0.472 to 0.145) and toward pH (0.329), thereby inverting the informational structure that entropy weighting imposes. The substantive conclusions are nevertheless invariant: the same four Brazilian basins remain embedded within the European range under every scheme, and Tietê-Alto and Tietê-Médio remain the two lowest-ranked entities in all four rankings. The ordinal conclusions of this study are therefore not an artefact of the entropy weighting.

The framework is designed to be transferable to any national monitoring network that can be linked to GRQA or an equivalent international archive, provided that sufficiently long observational records exist to estimate empirical improvement rates. The “External application to European monitoring networks” section tests this directly for the benchmarking and ranking components, which port successfully to Polish and Italian monitoring networks under a leave-one-country-out design. Further candidate networks include India (Central Pollution Control Board), China (National Environmental Monitoring Centre), and sub-Saharan Africa (UNESCO-IHP monitoring programmes); these remain untested here, as does the transferability of the convergence-planning component.

### External application to European monitoring networks

The framework is presented above as transferable to other national monitoring systems. Because that claim is central to the contribution, we tested it rather than asserting it, applying the framework to two European countries under a leave-one-country-out design and evaluating the result against an independent measure of anthropogenic pressure.

Poland and Italy were selected because they are the only countries in the GRQA extract with enough stations for basin-level aggregation (3885 and 3240 respectively) together with complete five-parameter coverage. For each in turn, the country’s monitoring stations were assigned to sub-basins using HydroBASINS level 6 (Lehner & Grill, 2013), and each basin was then treated exactly as the São Paulo basins are treated in the main analysis. Critically, the European reference distribution was recomputed with the target country *excluded*, so that no entity contributes to its own benchmark. Basins with fewer than 25 stations were dropped, leaving 33 Polish and 26 Italian basins.

Validating such an exercise requires a criterion that is external to the water quality data. Defining “clean” and “polluted” regions by hand would invite boundary drawing after the fact, so we instead used a continuous covariate produced by a different institution from unrelated inputs: basin population density from the European Commission Joint Research Centre Global Human Settlement Layer (GHS-POP R2023A, 1 km resolution, epoch 2020) (Pesaresi & Politis, 2023). The directional hypothesis was fixed in advance and is elementary: greater anthropogenic pressure should correspond to lower benchmarked water quality.

The prediction holds in both countries independently. Basin TOPSIS score correlates negatively with population density in Poland (Spearman’s ρ=-0.439, one-sided p=0.005, n=33) and in Italy (ρ=-0.424, p=0.015, n=26). That the association replicates across two hydrologically and industrially dissimilar settings, neither of which informed the development of the method, is the substantive result: applied to monitoring networks it was not built on, and benchmarked against a reference that excludes those networks, the framework orders basins in a way that tracks an independent measure of human pressure.

Two qualifications matter. First, the association is weighting-dependent in one of the two cases: it holds under entropy and CRITIC weights in both countries, but under equal weights it is significant in Italy (ρ=-0.373, p=0.030) and not in Poland (ρ=-0.215, p=0.115). Entropy weighting, which concentrates weight on the parameters that discriminate most strongly between entities, is evidently doing useful work here rather than merely reflecting an arbitrary choice — but the corollary is that the framework’s discriminating power is not wholly independent of how criteria are weighted, and we report it as such.

Second, the composite Wasserstein score shows no association with population density in either country (ρ=-0.074 and +0.050). This is a property of the measure rather than a failure of the test: $\overline{W}_1$ is an *unsigned* distance from the reference distribution, so a basin can be far from the European reference by being cleaner than it as well as by being more degraded. The composite should therefore be read as a measure of distributional dissimilarity and not as a quality ranking in its own right — a distinction that the parameter-specific $W_1$ values in Table 3 preserve but that the composite necessarily discards.

Finally, this exercise tests the benchmarking and ranking components only. The LP convergence model requires multi-decadal records at the basin level, which the GRQA extract does not provide for individual European basins, so the transferability of that component remains untested. We report the present result as a demonstration that the distributional and ranking machinery ports to other monitoring systems, not as a claim that the framework has been validated in full.

### Limitations

Several limitations should be noted. First, the comparison is restricted to the 2016–2022 window imposed by GRQA coverage, which does not capture the full temporal dynamics of the CETESB record; seasonal and inter-annual variability are aggregated in the distributional analyses, and future work should explore time-stratified benchmarking to capture seasonal dynamics explicitly.

The heterogeneity of the European pool deserves quantification rather than assertion, since it bears directly on whether a single pooled benchmark is meaningful. Between-country variation is large and statistically unambiguous for every parameter (Kruskal–Wallis $p < 10^{-300}$ in each case): country medians span 8.20–19.25 mg L$^{-1}$ for dissolved oxygen, 0.80–3.00 mg L$^{-1}$ for BOD$_5$, 0.005–0.283 mg L$^{-1}$ for total phosphorus, 0.004–3.099 mg L$^{-1}$ for nitrate, and 1.10–16.00 NTU for turbidity. A pooled European median is therefore a central tendency across genuinely dissimilar monitoring populations, not a description of a homogeneous reference condition.

The question this raises is whether the study’s conclusions depend on that pooling. They do not. Replacing the pooled benchmark with each individual European country in turn, the Brazilian median is worse than every one of the 17 countries reporting dissolved oxygen, all 19 reporting turbidity, 20 of 21 reporting total phosphorus, and 12 of 13 reporting BOD$_5$. The divergence is thus not an artefact of aggregating dissimilar countries: it holds against essentially any European country chosen as the comparator. Nitrate behaves differently, as expected from the main results — the Brazilian median exceeds only 10 of 19 country medians — which is precisely the parity finding reported in the “Results” section, now shown to be robust to the choice of reference rather than an averaging artefact.

Second, the European reference pools 23 countries with highly heterogeneous climatic, geographic, and monitoring characteristics. The inter-quartile range of the European pooled distribution of measurements reflects this heterogeneity: for dissolved oxygen, the p25–p75 range spans 8.00–11.20 mg L$^{-1}$; for total phosphorus, 0.028–0.120 mg L$^{-1}$; and for nitrate, 0.280–1.901 mg L$^{-1}$. The p50 benchmark used throughout this study therefore represents an aspirational central tendency rather than a uniform regulatory standard. As a sensitivity check, we re-ran the ranking analysis inserting a European reference entity constructed at three levels of stringency and recorded where each São Paulo basin falls relative to it. To keep the comparison on a single scale, the reference entity was built from quantiles of the distribution of European *country medians* — the same quantity that populates the decision matrix — rather than from quantiles of pooled individual measurements, and each quantile was oriented by criterion direction, so that a stricter reference means a higher value for dissolved oxygen and a lower value for the cost parameters. Under the most permissive reference (p75) one basin, Afluentes-PSB, scores above the reference; under the p50 and the demanding p25 references no Brazilian basin clears it, as expected when the comparator is drawn from the cleaner half and cleanest quartile of European rivers respectively. The internal ordering of the ten São Paulo basins is invariant across all three levels, with the single exception of Cantareira and Paraíba do Sul, which are separated by less than 0.001 in TOPSIS score and are best regarded as tied. The Tietê metropolitan arc (Alto and Médio) remains the lowest-ranked pair at every level. This monotonic and interpretable response to benchmark stringency — a steadily falling count of basins clearing the reference, rather than an erratic reshuffling — supports the robustness of the comparative conclusions despite the heterogeneity of the European pool.

**Table 9:** Sensitivity of critical-path convergence time $T_b^*$ to the percentile used to estimate the feasibility rate $r_j^{\max }$. The p90 column reproduces Table 7; the p75 column applies a more conservative feasibility frontier. Basins whose binding parameter changes between the two assumptions are marked †

Basin	$T_b^*$ p90	Binding p90	$T_b^*$ p75	Binding p75	Factor
	(yr)		(yr)		
Afluentes-PSB	4.4	DO	5.5	DO	1.25
Cantareira	4.9	DO	6.1	DO	1.24
Mogi-Guaçu	5.4	DO	6.6	DO	1.25
Jundiaí†	5.6	NO$_3^-$	12.3	TP	2.20
Paraíba do Sul	7.6	DO	9.4	DO	1.24
Capivari†	7.6	DO	12.3	TP	1.62
Piracicaba	7.8	DO	9.7	DO	1.24
Tietê-Baixo	11.6	NO$_3^-$	15.9	NO$_3^-$	1.37
Tietê-Médio	12.7	TP	34.2	TP	2.69
Tietê-Alto†	14.8	DO	28.7	TP	1.94

A related question is whether aggregating to medians conceals seasonal dynamics that would alter the comparison. Seasonality in the Brazilian record is real and, for some parameters, pronounced: comparing the wet season (October–March) with the dry season (April–September) over the analytical window, median turbidity is 26.95 versus 13.00 NTU (a factor of 2.07), total phosphorus 0.170 versus 0.110 mg L$^{-1}$ (1.55), dissolved oxygen 5.73 versus 6.30 mg L$^{-1}$ (0.91), and nitrate 0.720 versus 0.925 mg L$^{-1}$ (0.78), with Kruskal–Wallis p<0.01 in each case except BOD$_5$ (p=0.13). The wet-season elevation of turbidity and phosphorus is consistent with runoff-driven particulate and nutrient transport, and the dry-season elevation of nitrate with reduced dilution of a more continuous source.

Recomputing the entity ranking separately on wet-season and dry-season medians nevertheless leaves the comparative conclusions intact (Kendall’s τ=0.968 and 0.958 against the full-window ranking). Tietê-Alto and Tietê-Médio are the two lowest-ranked entities in both seasons, and Afluentes-PSB is the highest-ranked Brazilian basin in both; the only movement among the Brazilian basins is a reordering of Cantareira, Mogi-Guaçu and Paraíba do Sul, which are closely spaced in the full-window ranking in any case. Season therefore modulates the magnitude of the parameters without altering the structural conclusion. We note also that the Wasserstein and Mann–Whitney analyses operate on the full empirical distributions and so retain seasonal spread by construction; it is only the TOPSIS matrix and the LP gaps that aggregate to medians. Explicitly time-stratified benchmarking, including inter-annual and drought-year stratification, remains a priority for future work.

Third, the LP feasibility constraint uses the 90th-percentile annual improvement rate as a proxy for the maximum achievable rate. To quantify the uncertainty in the convergence-time projections, we re-solved the model with the more conservative 75th-percentile rate (Table 9). Convergence times lengthen for every basin, by factors ranging from 1.24 to 2.69. The most impaired systems are the most sensitive: Tietê-Médio moves from 12.7 to 34.2 years, Tietê-Alto from 14.8 to 28.7 years, Tietê-Baixo from 11.6 to 15.9 years, and Jundiaí from 5.6 to 12.3 years, whereas the low-burden basins shift only modestly (Afluentes-PSB from 4.4 to 5.5 years; Cantareira from 4.9 to 6.1 years).

A further result of this analysis is that the feasibility assumption affects not only the length of the planning horizon but the identity of the bottleneck. For three basins, the binding parameter changes under the p75 assumption: Jundiaí shifts from nitrate to total phosphorus, and Capivari and Tietê-Alto shift from dissolved oxygen to total phosphorus. This occurs because the feasibility rate for phosphorus falls proportionally further than that for oxygen when the percentile is lowered, so phosphorus overtakes oxygen in the gap-to-feasibility ordering. Convergence times should therefore be read as indicative planning horizons bracketed by these two assumptions rather than as point predictions, and the identification of a rate-limiting parameter should be understood as conditional on the assumed feasibility frontier. Real improvement trajectories may in addition be non-linear and subject to economic, regulatory, and infrastructure constraints that historical rates do not capture (Alizamir et al., 2025; Liang et al., 2025; Poursaeid, 2025; Wan et al., 2022).

Fourth, a substantial share of the Brazilian determinations for BOD$_5$ (41.2%) and, to a lesser degree, nitrate (14.3%) and total phosphorus (7.6%) are left-censored at the analytical detection limit and were used at the limit value. This inflates the Brazilian medians for those parameters and, with them, the BR/EU ratios and effect sizes reported in Tables 1 and 4. The bias is conservative for the conclusions drawn here — it makes the Brazilian network appear further from the European reference than it is — but it is real, and formal censored-data estimators such as regression on order statistics (Helsel et al., 2020) would be preferable where the censoring fraction is as high as it is for BOD$_5$. We flag this as a priority methodological refinement for future applications of the framework.

Fifth, the analysis is restricted to seven physicochemical parameters. Other ecologically relevant indicators — including biological indices, heavy metals, emerging contaminants, and microbiological parameters — are not included, and the conclusions regarding water quality should be understood in the context of this limited parameter set. Sixth, the study does not include biological indicators such as macroinvertebrate or phytoplankton indices, meaning that ecological status in the WFD sense is not directly comparable.

Regarding the statistical methods, non-normality of both datasets was confirmed via Shapiro-Wilk tests on random subsamples (n=5000) for each parameter: all parameters rejected normality at $p < 10^{-14}$ (e.g., BOD$_5$: BR $p = 3.4 \times 10^{-70}$, $\gamma _1 = 8.22$; EU $p = 1.6 \times 10^{-53}$, $\gamma _1 = 1.51$), with strongly right-skewed distributions and excess kurtosis confirming that parametric tests would be inappropriate. We note that, with samples of this size, a formal normality test will reject even mild departures from Gaussianity; the decisive evidence here is therefore not the *p*-value alone but the magnitude of the skewness and excess kurtosis, which place several parameters (BOD$_5$, TP, turbidity) far outside the range over which large-sample normal approximations remain reliable. This mirrors a recurring situation in large environmental datasets, where formal normality tests reject even negligible departures and analysts consequently rely on skewness and kurtosis to judge practical normality (Mehrabi et al., 2026); our data are more strongly non-normal than such borderline cases, reinforcing the choice of distribution-free methods. The Mann–Whitney U and Wasserstein-1 analyses are therefore methodologically justified, as both are distribution-free and robust to the heavy-tailed, right-skewed structure characteristic of surface water quality monitoring data (Helsel et al., 2020).

## Conclusions

This study applied a four-method framework — Wasser-stein-1 distributional distance, Shannon-entropy-weigh-ted TOPSIS, linear programming convergence planning, and PCA/Ward clustering — to benchmark ten São Paulo river basins against 23 European countries monitored under the Water Framework Directive, using 7 harmonised parameters over 2016–2022. The principal conclusions are:

1. São Paulo’s river quality is fundamentally bifurcated. Four basins (Afluentes-PSB, Paraíba do Sul, Cantareira, Mogi-Guaçu) rank within the European quality distribution and co-cluster at the clean-reference end of the ordination, while the Tietê metropolitan arc (Alto and Médio) ranks below all ten complete-coverage European countries as distributional outliers.
2. Wasserstein-1 distributional analysis reveals that the divergence between Afluentes-PSB and Tietê-Alto is an order of magnitude ($\overline{W}_1 = 0.245$ vs. 2.060), a heterogeneity that median comparison systematically underestimates.
3. Dissolved oxygen is the binding LP constraint for seven of ten basins, with critical-path convergence times ranging from 4.4 (Afluentes-PSB) to 14.8 years (Tietê-Alto) under the 90th-percentile feasibility assumption. Total phosphorus carries the highest LP cost weight (w=0.669) due to its low feasibility rate (0.074 mg L$^{-1}$ yr$^{-1}$), but is the rate-limiting parameter for only one basin (Tietê-Médio); nitrate is binding for two basins (Jundiaí and Tietê-Baixo). Both the timelines and the attribution of the binding constraint are conditional on the assumed feasibility frontier: under a more conservative 75th-percentile assumption, all convergence times lengthen, and phosphorus displaces oxygen or nitrate as the rate-limiting parameter in three basins. Convergence times should therefore be read as planning horizons bracketed by these assumptions rather than as forecasts.
4. Nitrate parity between Brazil and Europe is transient and mechanistically distinct: rising agricultural loading in the Piracicaba–Capivari–Jundiaí catchments is coinciding with European nitrate levels without the benefit of equivalent regulatory pressure, signalling an emergent eutrophication risk.
5. The comparative conclusions are robust to the principal analytical choices. The entity ranking is stable across alternative weighting schemes, including one that deliberately strips total phosphorus of its dominant entropy weight (Kendall’s τ=0.905 for equal weights and 0.863 for CRITIC), and the ordering of the São Paulo basins is invariant to the stringency of the European reference. What is *not* robust, and is reported as such, is the absolute convergence timescale and the identity of the rate-limiting parameter, both of which depend on the assumed feasibility frontier.
6. The benchmarking and ranking components of the framework port to other monitoring systems. Applied to 33 Polish and 26 Italian sub-basins under a leave-one-country-out design, with the European reference recomputed to exclude the target country, basin scores correlate negatively with an independent measure of anthropogenic pressure (population density from GHS-POP) in both countries (ρ=-0.439, p=0.005; ρ=-0.424, p=0.015). The framework also provides managerially actionable outputs — parameter-specific gaps, convergence horizons, basin priority tiers — that conventional index-based comparisons cannot generate. Transferability of the convergence-planning component, which requires multi-decadal basin-level records, remains untested and is a priority for future work.

The following governance implications are derived from the statistical findings presented above and are inten-ded as evidence-informed suggestions rather than prescriptive policy recommendations, which would require additional socioeconomic and institutional analysis. These findings support a differentiated governance architecture for São Paulo: investment concentration in the metropolitan Tietê corridor for structural DO and BOD$_5$ remediation, targeted phosphorus reduction in all basins, and proactive agricultural nitrate regulation in the UGRHI 5 and UGRHI 10 catchments before loading reaches European threshold levels.

From a monitoring network design perspective, the Wasserstein-1 composite score $\overline{W}_1$ proposed here constitutes a quantitative key performance indicator (KPI) for tracking how closely a monitored network’s distributional profile approaches a designated reference system over successive monitoring cycles. Unlike compliance rates or mean exceedance frequencies, $\overline{W}_1$ is sensitive to changes in distributional shape across all quantiles, providing an early-warning signal of deteriorating tail behaviour before threshold exceedances become prevalent. Monitoring agencies can use the parameter-specific $W_1$ decomposition (Table 3) to identify which physicochemical parameters contribute most to the distributional divergence and should therefore be prioritised in sampling frequency optimisation (Ying et al., 2025). The LP critical-path analysis further identifies the specific remediation rate required to achieve benchmark convergence within a target planning horizon, translating a distributional diagnostic into a directly actionable monitoring and management plan.

### Future research directions

The present analysis could be extended in several directions. First, expansion of the parameter set to include biological quality elements (macroinvertebrates, phytoplankton) and trace metals (e.g., As, Pb, Cd) would enable a more comprehensive ecological assessment. Second, alternative clustering algorithms — such as *k*-medoids or model-based clustering — could be explored to assess the sensitivity of the typological classification to the Ward linkage criterion. Third, the Wasserstein-LP framework could be applied at the within-basin, station-level scale to identify specific monitoring locations driving the aggregate distributional gaps identified here, directly informing sentinel station selection (Mehrabi et al., 2025; Ying et al., 2025).

## Supplementary Information

Below is the link to the electronic supplementary material.

Supplementary file 1 (zip 52 KB)

## Data Availability

The primary dataset used in this study is the CETESB QUALAR surface water quality database, maintained by the Companhia Ambiental do Estado de São Paulo and publicly accessible at https://cetesb.sp.gov.br/aguas-interiores/. The European benchmark dataset (GRQA v1.4) is openly available via Zenodo at https://doi.org/10.5281/zenodo.5097436 (Virro et al., 2021, Earth System Science Data, 13, 5483–5507). Processed annual compliance matrices and Python analysis code are available from the corresponding author upon reasonable request.
